# Hypoxia-induced reprogramming of the cardiac phenotype in American alligators (*Alligator mississippiensis*) revealed by quantitative proteomics

**DOI:** 10.1038/s41598-019-45023-3

**Published:** 2019-06-13

**Authors:** Sarah L. Alderman, Dane A. Crossley, Ruth M. Elsey, Todd E. Gillis

**Affiliations:** 1 Department of Integrative Biology, University of Guelph, Guelph, Ontario N1G 2W1 Canada; 2 Developmental Integrative Biology Research Group, Department of Biological Sciences, University of North Texas, Denton, Texas 76203-5017 USA; 3 Louisiana Department of Wildlife and Fisheries, Rockefeller Wildlife Refuge, Grand Chenier, Louisiana 70643 USA

**Keywords:** Reprogramming, Animal physiology, Heart development

## Abstract

Hypoxic exposure during development can have a profound influence on offspring physiology, including cardiac dysfunction, yet many reptile embryos naturally experience periods of hypoxia in buried nests. American alligators experimentally exposed to developmental hypoxia demonstrate morphological and functional changes to the heart that persist into later life stages; however, the molecular bases of these changes remain unknown. We tested if targeted and persistent changes in steady-state protein expression underlie this hypoxic heart phenotype, using isobaric tags for relative and absolute quantitation (iTRAQ) proteomics. Alligator eggs were reared under normoxia or 10% hypoxia, then either sampled (embryo) or returned to normoxia for 2 years (juvenile). Three salient findings emerge from the integrated analysis of the 145 differentially expressed proteins in hypoxia-reared animals: (1) significant protein-protein interaction networks were identified only in up-regulated proteins, indicating that the effects of developmental hypoxia are stimulatory and directed; (2) the up-regulated proteins substantially enriched processes related to protein turnover, cellular organization, and metabolic pathways, supporting increased resource allocation towards building and maintaining a higher functioning heart; and (3) the juvenile cardiac proteome retained many of the signature changes observed in embryonic hearts, supporting long-term reprogramming of cardiac myocytes induced by hypoxia during critical periods of development.

## Introduction

Developmental plasticity is the process by which phenotypic variation in offspring is induced by environmental variables, and its contribution to everything from evolutionary processes^1^ to human disease pathology^2–5^ is widely recognized. Hypoxia is among the most potent drivers of developmental plasticity in vertebrates, owing to the obligatory role of oxygen in cellular metabolism^1,6,7^. Considering that the cardiovascular system is the first functional organ system in developing vertebrates^8^, the high metabolic demand of cardiac tissue, and the primary convective function of this system for delivering oxygen to peripheral tissues, it is perhaps not surprising that hypoxic exposure during early development can impart significant and lasting effects on the vertebrate heart. In placental mammals, where oxygen availability for the developing fetus varies little under normal circumstances, gestational hypoxia is tightly coupled to increased risk for hypertension and cardiovascular disease later in life^2–5^. In contrast, many oviparous reptiles bury large clutches of eggs in subterranean or mound nests that can limit potential gas exchange, and so embryonic development naturally occurs in an environment where oxygen tension can vary considerably^9,10^. This natural history trait presents a unique opportunity to study the potentially adaptive outcomes of hypoxia-induced developmental plasticity.

The cardiac phenotype of embryonic reptiles reared under low-oxygen conditions is characterized by hypertrophy^11–13^, reduced normoxic heart rate^11^, and the ability to maintain cardiac function during acute hypoxic exposure^11,14^. The critical window for inducing this cardiac phenotype occurs early in embryonic development, and is distinct from the window for hypoxia-induced somatic growth restriction^15,16^. Recent longitudinal studies of reptiles that were exposed to hypoxia during embryonic development and then returned to normoxia after hatching, suggest that some of these phenotypic responses are maintained into later life stages. For example, Wearing *et al*.^17^ used feeding after a period of fasting to increase demand on the cardiovascular system, and quantified metabolic rates of 3 y old snapping turtles that were previously exposed to developmental hypoxia. Compared to normoxia-reared turtles, juvenile turtles exposed to developmental hypoxia had lower heart rates, higher post-feeding metabolic rates, and reached peak post-prandial metabolic rate sooner^17^. The heightened metabolism of hypoxia-reared turtles during this period of increased oxygen demand coincided with an increased capacity to divert blood flow through the left-to-right shunt into the systemic circuit^18^. In another study, Joyce and colleagues^19^ reported that the increase in relative heart mass of alligators exposed to developmental hypoxia is maintained in 4 y old juveniles, and also demonstrated increased β-adrenergic sensitivity during exercise in these animals^19^. Combined, these studies provide compelling evidence that developmental hypoxia exposure reprograms heart morphology and function to augment performance under conditions where oxygen is limiting (ex. breath-holding during diving), and is thus an example of adaptive developmental plasticity. It remains unknown how these changes are implemented and maintained at the molecular level, and insight here may contribute to a deeper appreciation for the regulatory differences between physiological and pathological cardiac programming.

The aim of the present study was to identify the protein signatures that underpin the cardiac phenotype of hypoxia-reared alligators, and to determine if long-term changes in steady-state protein expression help maintain this phenotype into juvenile life stages. In models of exercise, disease states, ageing, and other physiological conditions, changes in the muscle proteome are closely related to observed differences in muscle function^20^. Therefore, we used quantitative shotgun proteomics to simultaneously identify and quantify the relative abundance of hundreds of proteins in the hearts of embryonic and juvenile alligators that were either reared continuously in normoxia, or were exposed to hypoxia during early development. This powerful, comprehensive approach revealed a suite of targeted changes in protein abundance induced by hypoxia that occur during embryonic development and are carried forward into later life stages. These protein expression changes contribute to a significant enrichment of functional pathways related to protein turnover, cellular organization, and cellular metabolism, and these findings are discussed in relation to increased performance of the heart in hypoxia.

## Results

### Cardiac proteome characterization

**Figure 1:**
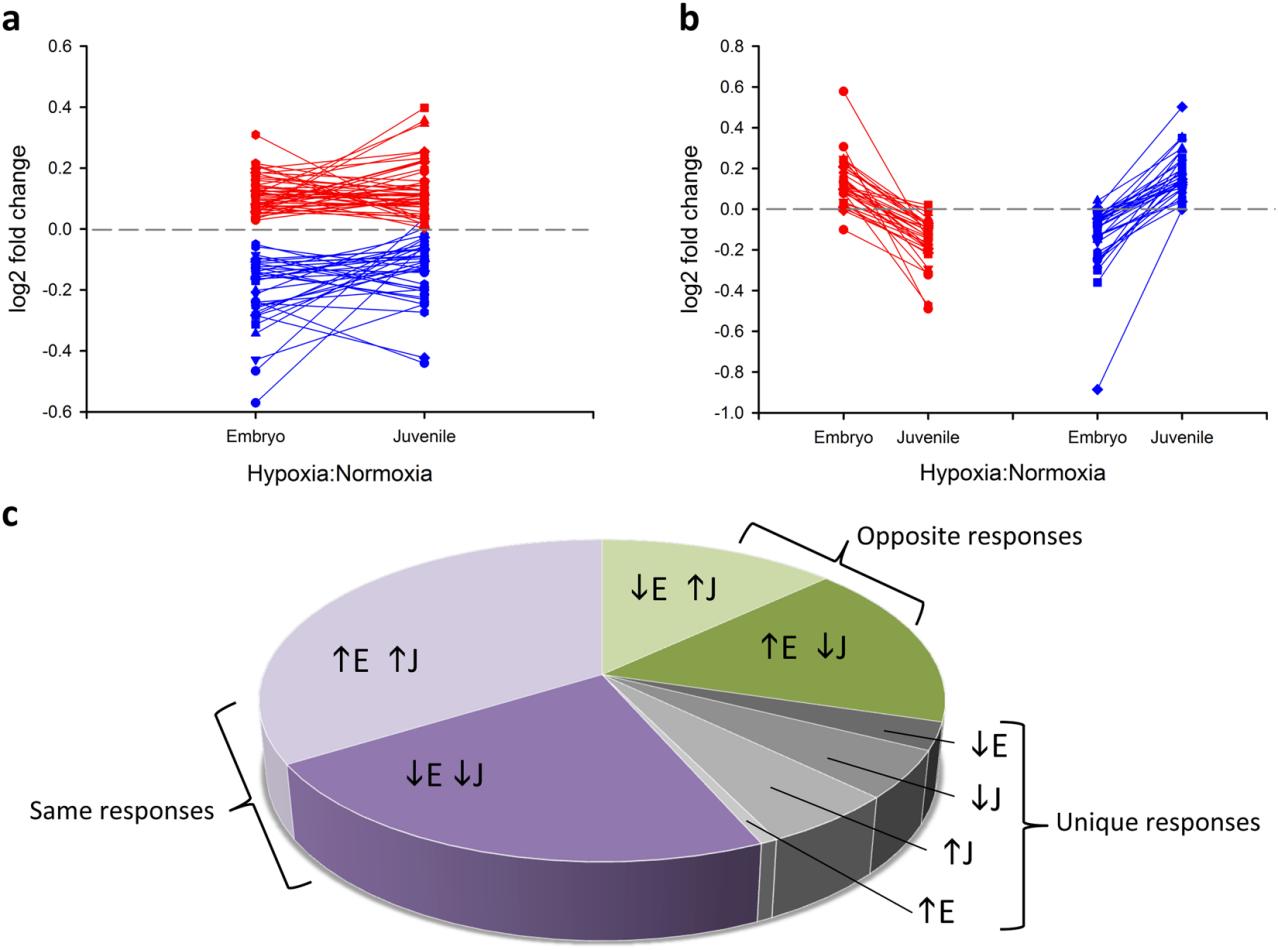
Patterns of protein expression changes induced by developmental hypoxic exposure in embryonic (E, 90% incubation) and juvenile (J, 2 y old) alligator hearts. (**a**) Differentially abundant proteins with p < 0.05 for the main effect of *oxygen*, where red are up-regulated and blue are down-regulated proteins, expressed as log2 fold change from the normoxic controls. (**b**) Differentially abundant proteins with p < 0.05 for the interaction effect of *oxygen* × *age*, where red traces are proteins with higher expression in embryonic than juvenile hearts, and blue traces indicate the opposite trend, in log2 fold change from normoxic controls. (**c**) Proportional representation of the 145 differentially abundant proteins, grouped as those with similar direction of response in embryonic and juvenile hearts (purple sections), those with opposite direction of response between the two ages (green sections), and those proteins that were uniquely regulated in a single treatment (grey sections).

The alligator ventricular proteome was described by a total of 904 high quality and high confidence protein assignments that were identified in all samples and by at least two unique peptides. The abundances of 557 proteins were significantly altered by *age* (509 proteins), *oxygen* (97 proteins), or their *interaction* (*age* × *oxygen*; 65 proteins). In keeping with our main objective of determining the impacts of developmental hypoxia exposure on the cardiac proteome, only protein expression changes related to *oxygen* (Fig. 1a) and *interaction* (Fig. 1b) are considered herein.

### Effects of developmental hypoxia

Representative lists of differentially abundant proteins are provided for *oxygen* (Table 1) and for *interaction* (Table 2), with full details available in (Supplementary Data S1). In general, the magnitude of change was small. Of the 97 proteins significantly altered by *oxygen*, 8 proteins changed by more than 1.2 fold-change (FC) and no proteins had expression changes exceeding 1.5 FC. Similarly, of the 65 proteins significantly altered by the *interaction* term, 15 proteins were above 1.2 FC and 2 proteins exceeded 1.5 FC. To compare both common and age-specific responses, and to maximize input for functional analyses, differentially abundant proteins for *oxygen* and *interaction* were combined to yield 72 or 79 up-regulated proteins, and 59 or 64 down-regulated proteins in embryonic or juvenile hearts, respectively (145 unique proteins in total). More than half of the proteins altered in embryonic hearts were similarly altered in the juvenile hearts (66% and 56% similarity for up- and down-regulated proteins between ages, respectively; Fig. 1c). In contrast, roughly one third of proteins showed oppositional changes in abundance between ages, and only a handful of proteins were uniquely regulated in a single experimental group (Fig. 1c). A protein with oppositional changes, Natriuretic Peptide A (nppa), was used to cross-validate the iTRAQ results using an orthogonal method (qRT-PCR). As with protein abundance, mRNA abundance for *nppa* was increased by developmental hypoxia in embryonic hearts, and decreased by developmental hypoxia in juvenile hearts, with a strong correlation between gene and protein expression across all treatments (R^2^ = 0.76625; Supplementary Fig. S1).

**Table 1:** Differentially abundant proteins for the main effect *oxygen*, where p < 0.05 and log2FC > |0.2|.

Gene	Description	Function	log2FC	*p*
***Up-regulated***				
COPS2	COP9 signalosome complex subunit 2	regulates protein degradation	0.274	0.016
COX2	Cytochrome oxidase subunit II	oxidative phosphorylation	0.240	0.024
ARHGDIA	Rho GDP-dissociation inhibitor 1	regulates GTPase signalling	0.226	0.017
PSMD4	Proteasome 26S subunit 4	degradation of ubiquitinated proteins	0.217	0.035
MAIP1/C2orf47	Matrix AAA peptidase interacting protein 1	mitochondrial protease	0.209	0.031
MRPS29/DAP3	Mitochondrial 28S ribosomal protein	translation; apoptosis	0.203	0.030
YWHAB	14-3-3 alpha	intracellular signalling	0.200	0.020
***Down-regulated***				
FIS1	Mitochondrial fission protein 1	regulates mitochondrial morphology	−0.396	0.015
(KYO27548.1)	No human ortholog	unknown	−0.377	0.026
CRK	Adapter molecule crk	intracellular signalling	−0.365	0.021
PPM1E	Protein phosphatase 1E	protein dephosphorylation, including CAMK	−0.340	0.032
DYNC1LI2	Dynein cytoplasmic 1 intermediate light chain 2	microtubule-associated motor protein	−0.324	0.045
SMS	Spermine synthase	catalyzes spermine production	−0.304	0.045
STX12	Syntaxin-12	regulates protein transport	−0.265	0.034
RTN1	Reticulon-1	membrane trafficking	−0.243	0.028
CDK5RAP3	CDK5 regulatory subunit-associated protein 3	transcriptional regulation, cell cycle progression	−0.242	0.020
GPD1L	Glycerol-3-phosphate dehydrogenase 1-like	regulates cardiac sodium current	−0.232	0.031
TPMT	Thiopurine S-methyltransferase	thiopurine metabolism; endogenous function unknown	−0.211	0.011
NRAP	Nebulin related anchoring protein	myofibrilar organization in cardiomyocytes	−0.210	0.033
COA3	Cytochrome c oxidase assembly factor 3	mitochondrial assembly	−0.207	0.034
SAMHD1	Deoxynucleoside triphosphate triphosphohydrolase	regulates dNTP pool	−0.204	0.001
ADHFE1	Hydroxyacid-oxoacid transhydrogenase, mitochondrial	oxidoreductase activity	−0.202	0.003

Up-regulated and down-regulated proteins are presented separately, ordered by magnitude of log2FC. Where no human homolog was found and function was unknown, protein is listed by Accession number in place of gene symbol. FC = fold change.

**Table 2:** Differentially abundant proteins for the Interaction term *age* × *oxygen*, where p < 0.05 and log2FC > |0.2|.

Gene	Description	Function	log2FC 90%	log2FC J	*p*
EIF5A2	Eukaryotic translation initiation factor 5A-2	translation	**−0.250**	0.139	0.001
ENO2	Enolase 2	glycolysis	**0.307**	**−0.490**	0.002
COPS3	COP9 signalosome complex subunit 3	regulates protein degradation	−0.050	**0.353**	0.003
SEC.11A	Signal peptidase complex catalytic subunit	removes signal peptides from nascent proteins	**0.207**	−0.081	0.005
OVOS	Ovostatin homolog	proteinase inhibitor	**−0.275**	**0.293**	0.006
GDA	Guanine deaminase	purine metabolism	**−0.213**	0.018	0.008
SGCB	Beta-sarcoglycan	links muscle cytoskeleton to extracellular matrix	0.007	**0.502**	0.008
PLD3	Phospholipase D3	hydrolyzes membrane phopsholipids	0.132	**−0.323**	0.008
IGSF9	Ig superfamily member 9	cell adhesion	**0.242**	−0.080	0.014
TFRC	Transferrin receptor	cellular iron uptake	**0.208**	−0.068	0.014
EHD2	EH domain containing 2	endocytosis	0.042	**0.298**	0.014
SOD3	Cu-Zn superoxide dismutase	antioxidant	−0.150	**0.290**	0.014
NPPA	Natriuretic peptide A	regulates extracellular fluid volume	**0.248**	**−0.293**	0.015
CD34	Hematopoietic progenitor cell antigen	cell adhesion	**0.578**	−0.151	0.017
PSMB3	Proteasome subunit beta type-3	degradation of ubiquitinated proteins	**0.245**	−0.044	0.017
ESD	S-formylglutathione hydrolase B	serine hydrolase	**−0.252**	0.069	0.017
PYGB	Glycogen phosphorylase	glycogen mobilization	**−0.235**	0.098	0.018
MYO1C	Unconventional myosin-lc	intracellular movement	−0.066	**0.242**	0.021
TPD52L2	Tumor protein D54	cell proliferation	0.024	**−0.221**	0.021
THYN1	Thymocyte nuclear protein	induction of apoptosis	0.085	**−0.473**	0.025
VCAN	Versican core protein	cell adhesion	0.224	−0.101	0.026
DCPS	Decapping scavenger enzyme	mRNA degradation	**−0.885**	−0.001	0.030
SGCD	Sarcoglycan delta	links muscle cytoskeleton to extracellular matrix	−0.027	**0.229**	0.030
GRSF1	G-rich sequence factor 1	regulates mitochondrial gene expression	−0.105	**0.349**	0.035
CMAS	N-acylneuraminate cytidylyltransferase	sialic acid synthesis	**−0.248**	0.143	0.040
ARL6IP5	PRA1 family protein 3	regulates intracellular taurine and glutamate	**−0.300**	**0.207**	0.040
CLYBL	Citrate lyase subunit beta	mitochondrial vitamin b12 metabolism	**−0.360**	**0.252**	0.041
NDUFAF6	NADH:ubiquinone oxidoreductase assembly factor	complex I biosynthesis	0.011	**−0.215**	0.044
SAMHD1	Deoxynucleoside triphosphate triphosphohydrolase	innate immune response	−0.101	**−0.314**	0.047

Proteins are listed by increasing p-value, and significant log2FC fields are in bold font. FC = fold change; 90% = 90% incubation; J = juvenile.

### Pathway analysis

**Figure 2:**
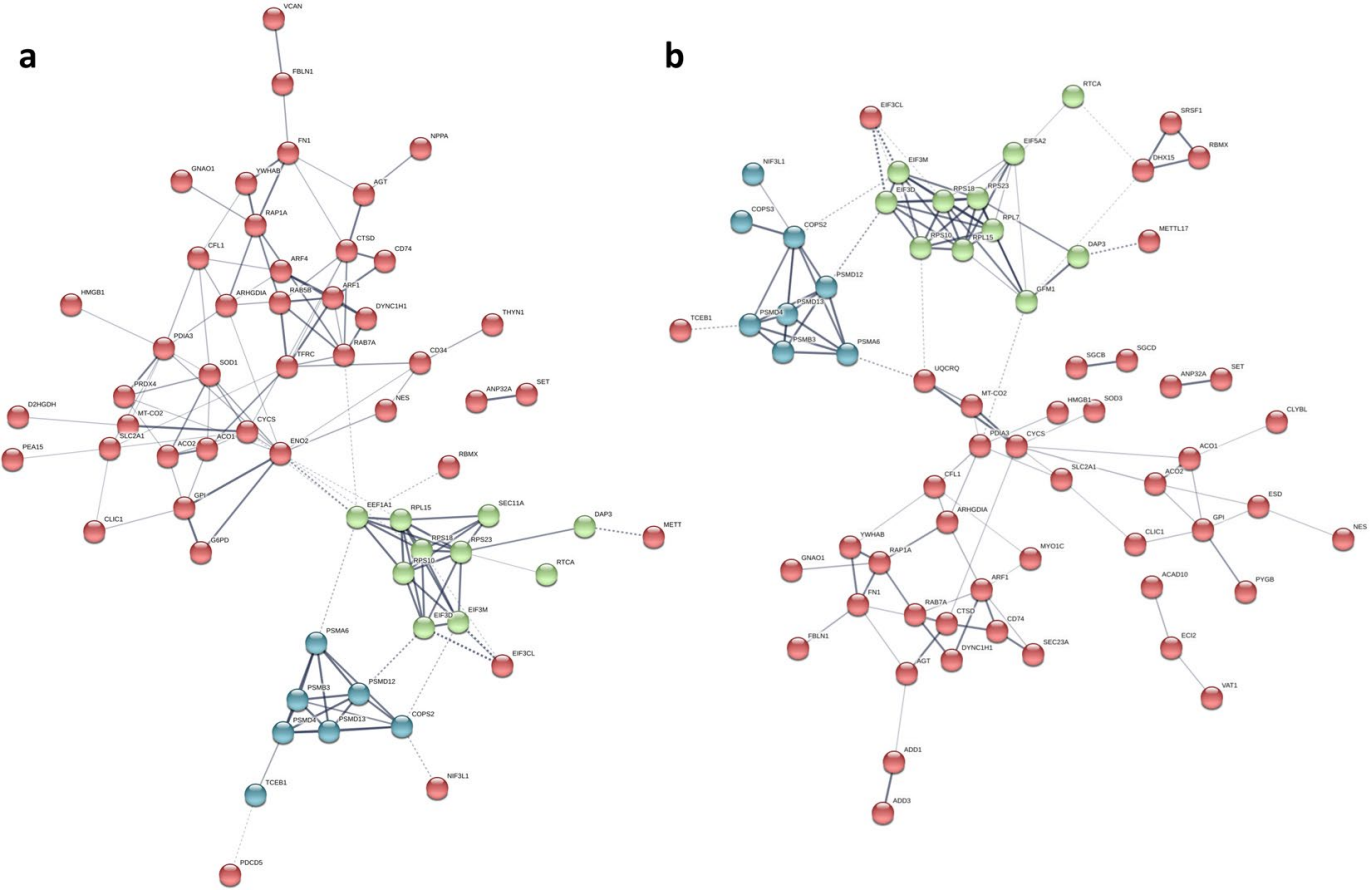
Significant protein-protein interaction networks for proteins up-regulated in the heart ventricles of (**a**) 90% incubation and (**b**) juvenile alligators exposed to hypoxia during development (p-values 8.39e-13 and 8.1e-9, respectively). Interactions were assigned at default confidence level (0.400), and disconnected nodes were hidden. The network map was grouped into 3 clusters using K-means method in STRING (https://string-db.org/).

The up- and down-regulated proteins for embryonic and juvenile hearts were analyzed separately in STRING to determine functionally enriched pathways. Significant protein-protein interaction networks were evident only for the up-regulated proteins (*P*-values 8.39 × 10^−13^ and 8.1 × 10^−9^, for embryo and juvenile, respectively; Fig. 2). Results are further described along the three primary GO categories, as well as by KEGG pathway enrichment.

#### Biological process

**Figure 3:**
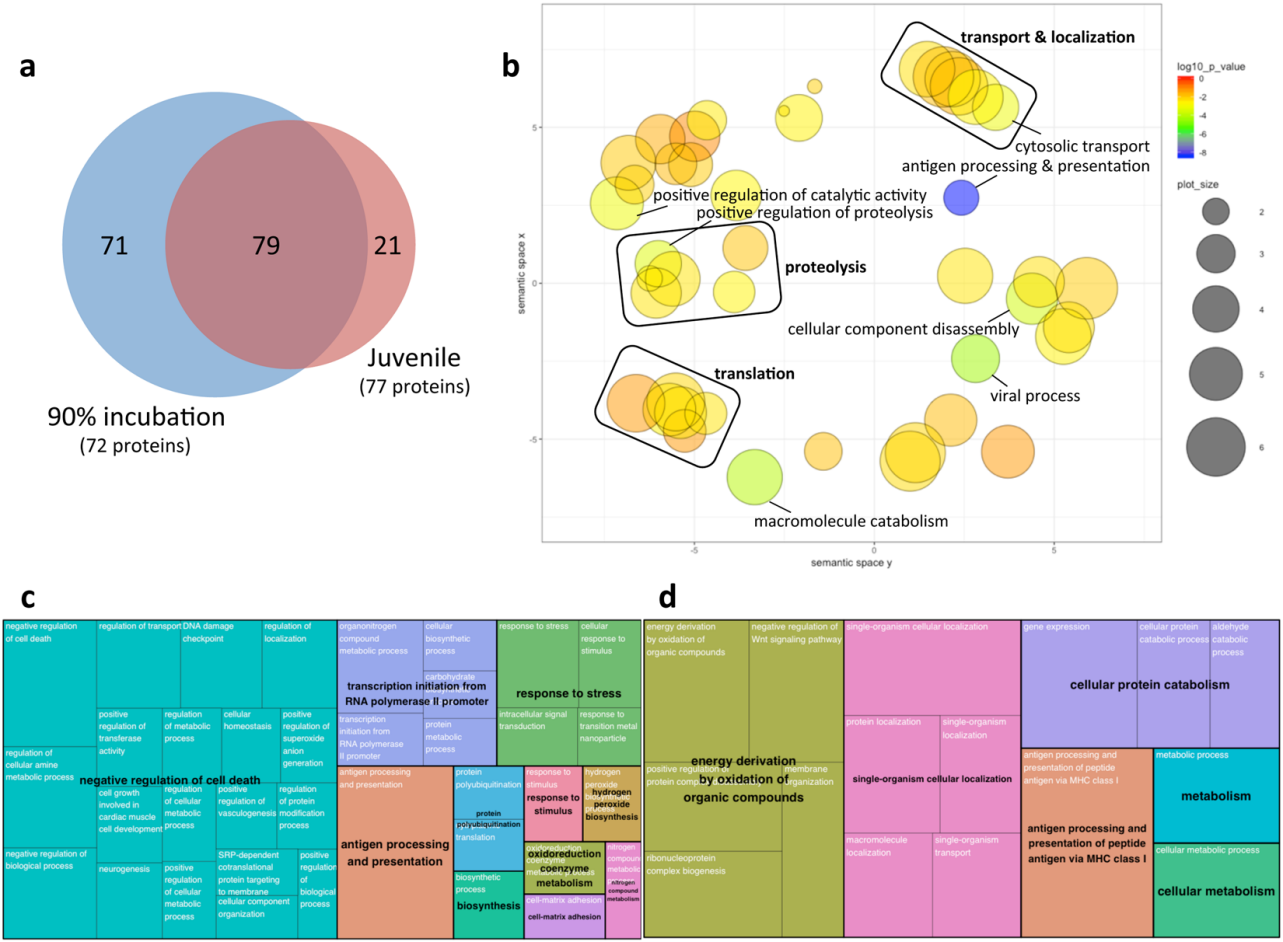
Visualizations of Gene Ontology (GO) functional enrichment for Biological Process. GO assignments were performed for 72 (90% incubation) or 77 (juvenile) proteins up-regulated by developmental hypoxia exposure using STRING (https://string-db.org/), yielding 150 and 100 significantly enriched GO terms for Biological Process, respectively. (**a**) Venn diagram depicting the similarity of GO term enrichment at each developmental stage. (**b**) Semantic plot of the 79 GO terms commonly enriched by hypoxia for each developmental stage, generated using default settings in REVIGO (http://revigo.irb.hr/) to depict the similarity among non-redundant GO terms. Bubble color indicates the log10(p-value) for the false discovery rates determined in STRING. Bubble size indicates the frequency of the GO term in the protein annotation database (whole UniProt), where bubbles of more general terms are larger. Individually labeled bubbles are equivalent to p < 0.001. (**c**,**d**) Treemap summaries for the 71 and 21 uniquely enriched GO terms at 90% incubation and juvenile stage, respectively, generated using default settings in REVIGO. Each box represents a cluster of redundant GO terms (cluster name in white font), with box size adjusted to p-value and box colour indicating super-clusters of loosely related terms (super-cluster name in black font).

There was a profound up-regulation of proteins associated with Biological Process induced by developmental hypoxia exposure. A total of 150 and 100 GO terms were significantly enriched by hypoxia exposure in embryonic and juvenile stage hearts, respectively, and more than half of these GO terms were common to both ages (Fig. 3a; Supplementary Data S1). The functional significance of these changes was explored using the non-biased GO visualization and redundancy reduction tool ReviGO^21^. The 79 GO terms commonly enriched by hypoxic exposure in embryonic and juvenile hearts were highly clustered to processes related to cellular transport and localization, protein turnover (including proteolysis and translation), cellular organization, as well as several catalytic and immune system related processes (Fig. 3b). The 71 GO terms uniquely enriched by hypoxia in embryonic hearts clustered to processes related to the negative regulation of cell death, transcription, cellular response to stress, as well as several biosynthetic and metabolic processes (Fig. 3c). The 21 GO terms uniquely enriched by hypoxia in juvenile hearts clustered to processes related to energy production and metabolism, cellular localization, protein catabolism, and antigen processing (Fig. 3d). For perspective, only a single GO term under Biological Process was enriched by down-regulated proteins, and only in juvenile hearts (14 proteins grouped to oxidation-reduction process, GO:0055114); however, this pathway was also enriched by up-regulated proteins in both embryonic (16 proteins) and juvenile hearts (13 proteins).

#### Cellular component

**Figure 4:**
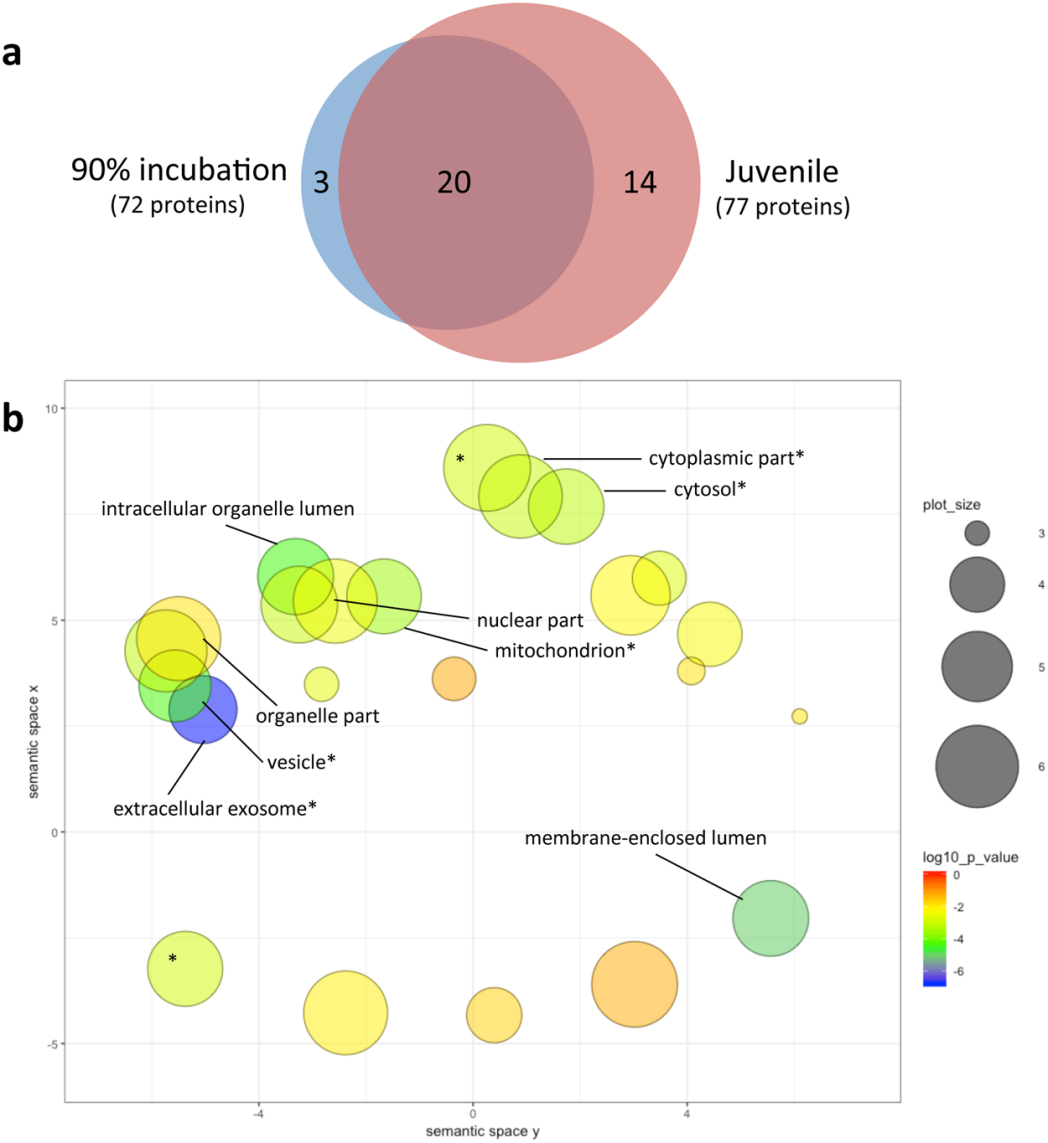
Visualizations of Gene Ontology (GO) functional enrichment for Cellular Component. GO assignments were performed for 72 (90% incubation) or 77 (juvenile) proteins up-regulated by developmental hypoxia exposure using STRING (https://string-db.org/), yielding 23 and 34 significantly enriched GO terms for Cellular Component, respectively. (**a**) Venn diagram depicting the similarity of GO term enrichment at each developmental stage. (**b**) Semantic plot depicting similarity of non-redundant GO terms enriched by hypoxia in juvenile hearts, generated using default settings in REVIGO (http://revigo.irb.hr/). Bubble color indicates the log10(p-value) for the false discovery rates determined in STRING. Bubble size indicates the frequency of the GO term in the protein annotation database (whole UniProt), where bubbles of more general terms are larger. Individually labeled bubbles are equivalent to p < 0.001, and an asterisk indicates a GO term also enriched by hypoxia at 90% incubation.

Pathway enrichment for proteins associated with Cellular Component ranged from 15 to 34 GO terms, depending on age and direction of change (Supplementary Data S1). Several GO-terms were enriched in both up-regulated and down-regulated proteins in each group of animals, including extracellular exosome (GO:0070062), mitochondrion (GO:0005739), and cytosol (GO:0005829). Given that significant PPI occurred only in up-regulated proteins, and that only 3 of the 15 GO terms were uniquely enriched in embryonic hearts (blood microparticle GO:0072562; extracellular space GO:0005615; myelin sheath GO:0043209; Fig. 4a), visualization of GO enrichment for Cellular Component was performed for up-regulated proteins in juvenile hearts only. These 34 GO terms were highly clustered, indicating considerable similarity among terms, including clusters related to cytoplasmic components and to organelle/organelle part components (Fig. 4b).

#### Molecular function

Developmental hypoxic exposure had little discernable effect on pathways associated with Molecular Function. A total of 8 GO terms were enriched among all 4 comparisons of up- and down-regulated proteins at each age (Supplementary Data S1). Half of the GO terms clustered to functions related to translation, and these were down-regulated in embryonic hearts and up-regulated in juvenile hearts.

#### KEGG pathways

Developmental hypoxic exposure also had little impact on KEGG pathway enrichment (7 pathways in total; Supplementary Data S1). Included among these were Proteasome (3050; enriched by up-regulated proteins in both embryonic and juvenile hearts) and HIF-1 signaling pathway (4066; up-regulated in embryos and down-regulated in juveniles).

## Discussion

Hypoxia is a major driver of phenotypic change during development. There is considerable support from studies with mammalian models that link gestational hypoxia to the pathogenesis of disease in later life^2,3^, including cardiovascular dysfunction^4,5^, however the mechanisms underlying such responses are not well defined. In reptiles, too, developmental hypoxic exposure alters the morphology and function of the embryonic heart^11,15,16,22^, and phenotypic differences remain intact years after hatching^12,17–19^. Importantly, improved performance of the cardiovascular system under subsequent hypoxic exposure^11,23^ or increased cardiac demand^17–19^ suggests that, unlike in placental mammals, developmental hypoxic exposure may impart beneficial changes to the cardiac phenotype of reptiles, who may naturally experience a low oxygen environment in subterranean nests^9^. Our study provides novel, comprehensive data on the molecular origins of this phenotypic change, using shotgun proteomics to quantitate differences in steady-state protein abundances between hypoxia- and normoxia-reared alligators. We show that developmental hypoxic exposure induces a substantial shift in the cardiac proteome, and identify protein synthesis (transcription and translation), cellular organization, metabolic adjustments, and protein degradation, as primary signatures of the hypoxia-induced cardiac phenotype. Importantly, these protein signatures are in place prior to hatching and are largely maintained into the juvenile stage, suggesting a lasting increase in the heart’s capacity to synthesize, utilize, and recycle proteins. These findings are considered in the contexts of cardiac hypertrophy and metabolic reprogramming, with perspectives on how these proteomic signatures may contribute to supporting cardiac function when oxygen is limiting.

### Developmental hypoxia induces cardiac hypertrophy

Environmental hypoxic exposure during reptile embryogenesis is known to restrict somatic growth and differentially increase the relative mass of other organs, including the heart^11^. The critical window for cardiac enlargement precedes, and is distinct from, the critical window for somatic growth restriction^15,16^, supporting cardiac hypertrophy as a targeted and direct response to hypoxia, as opposed to a non-specific outcome of reduced somatic growth. The present study adds to this interpretation by revealing significant changes to the cardiac proteome of hypoxia-reared alligators relative to normoxia-reared cohorts. Specifically, 16% of the identified cardiac proteome was altered by developmental hypoxia, and this response was over-and-above a considerable age-related effect on the cardiac proteome. While the number of increasing and decreasing proteins resulting from developmental hypoxia exposure was similar (55% and 45%, respectively), significant PPI networks were only identified for the up-regulated proteins. Similarly, functional enrichment of GO terms was heavily skewed towards up-regulated proteins (e.g. 100 vs. 1 Biological Processes GO term for up- and down-regulated proteins in juvenile hearts, respectively). This suggests that the hypoxia stimulus drives concerted changes in the relative expression of cardiac proteins to specifically enhance key cellular pathways, and warrants further study into the possible epigenetic mechanisms at play that enable this persistent increase in target protein expression. At the same time, the absence of network formation for down-regulated proteins could be interpreted as a null response with little functional relevance, which is further supported by the limited functional enrichment of GO terms by these proteins. As discussed later, however, down-regulation of certain proteins may be an important counter measure to re-allocate energy budget and cellular resources (ex. amino acid pool) away from non-essential pathways.

Functional analysis of the proteins up-regulated by developmental hypoxic exposure revealed substantially more pathway enrichment within Biological Processes as opposed to the other two primary GO terms (Cellular Component, and Molecular Function), including considerable emphasis on pathways related to protein synthesis and cellular organization. For example, numerous proteins involved in transcription and translation were up-regulated in embryonic and/or juvenile hearts exposed to developmental hypoxia, including certain mitochondrial-specific translation factors (e.g. mitochondrial 28S ribosomal protein, DAP3; G elongation factor mitochondrial 1, GFM1). Even after accounting for redundancy in pathway terms, transcription and translation remained a prominent feature of the pathway analysis within Biological Process (Fig. 3). Moreover, cellular transport and localization was also a discernible feature of the pathway analysis, which indicates that the capacity for organizing these newly made proteins within the cell is simultaneously increased alongside protein synthesis. These changes are easily understood as a prerequisite to support cardiac enlargement. In addition, this increased capacity to make and integrate new proteins may support enhanced protein turnover as a mechanism to circumvent the accumulation of damaged proteins (see below).

Contractile proteins were not among the differentially abundant proteins identified in this study. This suggests that the relative abundance of contractile proteins, or the number of contractile units compared to other cellular components, has been maintained. However, it is also quite likely, considering the extended experimental timeline of the present study, that a transient increase in contractile protein abundance needed to support the initial hypertrophic response was missed. In rainbow trout, for example, an exercise-induced increase in the myofilament protein, troponin I, was apparent at 4 d of training but returned to baseline by 7 d of training^24^. In the current study, however, hypoxia-induced increases in other structural proteins was observed (e.g. cofilin 1, CFL1; elastin microfibril interfacer 2, EMILIN2; nestin, NES; dynein cytoplasmic 1 heavy chain 1, DYNC1H1; unconventional myosin light chain, MYO1C in juvenile hearts) and cell adhesion molecules (e.g. in embryos: hematopoietic progenitor cell antigen, CD34, and versican core protein, VCAN; in juveniles: sarcoglycans B and D, SGCB and SGCD) which support a shift towards increased cell and tissue integrity. Such changes would help provide structural support to the myocardium as the force generating capacity of the heart increases during hypertrophy.

### Developmental hypoxia alters metabolic protein abundance

A distinguishing feature between physiological and pathological cardiac plasticity in mammals is a switch in metabolic fuel preference. Unlike in skeletal muscle, lipids are a preferred substrate for energy production in the heart^25^. With exercise, the heart’s capacity for fatty acid oxidation and the TCA cycle are increased to support a greater dependence on oxidative phosphorylation for energy and to avoid deleterious effects of lipid accumulation in cardiomyocytes^20,26,27^. In contrast, in a guinea pig model of hypertension-induced heart failure, integrated multi-platform “omics” analyses revealed broad suppression of TCA cycle enzyme abundances, and the subsequent accumulation of long-chain fatty acids marked the transition from hypertrophy to heart failure in these animals^28^. In alligator hearts exposed to developmental hypoxia, we found increased abundances of proteins associated with fatty acid oxidation (e.g. acyl-CoA dehydrogenase family member 10, ACAD10; acyl-CoA thioesterase 2, ACOT2, and enoyl-CoA delta isomerase 1, ECI2 in juvenile hearts), the TCA cycle (e.g. aconitase 1 and 2), and oxidative phosphorylation (e.g. cytochrome oxidase subunit II, COX2; cytochrome C somatic, CYCS). In addition, these and other up-regulated proteins enriched numerous GO pathways related to cellular metabolism, including specific (e.g. oxidation-reduction process, GO:0055114) and more general (e.g. cellular metabolic process, GO:0044237) terms, supporting the overall conclusion that developmental hypoxia exposure reprograms alligator hearts to increase metabolic capacity. Also worth noting is the reverse regulation of the glycolytic enzyme, enolase 2 (ENO2), and the rate-limiting enzyme in the pentose-phosphate pathway, glucose-6-phosphate dehydrogenase (G6PD). Both of these proteins were up-regulated by hypoxia in embryonic hearts but down-regulated in juvenile hearts, implying increased reliance on carbohydrate fuel sources during embryonic development when the hypoxia stressor was still present, followed by a significant shift away from glucose metabolism as the animals continued to develop in a normoxic environment.

Oxidative phosphorylation in tissue mitochondria is the backbone of ATP production in aerobic metabolism, where oxygen is used as the final electron acceptor from COX2 in the electron transport chain. When oxygen is in short supply, numerous morphological and functional changes to mitochondria represent an important component of the physiological response to hypoxia. Surprisingly, Galli and colleagues reported that mitochondrial function in the hearts of embryonic alligators was largely unchanged by developmental hypoxia in embryos; however, lower leak respiration rates were observed in the hearts of juvenile alligators that were exposed to developmental hypoxia, suggesting that the long-term changes in cardiac phenotype of hypoxia-reared reptiles include increased mitochondrial efficiency^12^. The authors posit that alterations to mitochondrial membrane composition and/or expression of uncoupling proteins could enable the observed reduction in proton leak, and question whether increased oxidative damage – as a consequence of improved mitochondrial efficiency – poses a long-term risk to cardiac health^12^. Our results indicate that the abundances of some uncoupling proteins are indeed modified by developmental hypoxia exposure (e.g. COX2, CYCS); however, increased capacity for free radical scavenging was not well supported at the protein level. Specifically, while the abundance of Cu-Zn superoxide dismutase (SOD3) was higher in juvenile hearts previously exposed to hypoxia, other antioxidants were down-regulated in these hearts (e.g. Peroxiredoxin 4 and SOD1). Nor was there any indication of a constitutive increase in heat-shock proteins, which accompanies the suite of adaptations in anoxia-tolerant turtles^29^. We therefore propose an alternate hypothesis that could help hypoxia-exposed alligator hearts circumvent an accumulation of oxidized proteins. Functional enrichment of proteolytic pathways was a key feature in both embryonic and juvenile hearts of alligators exposed to developmental hypoxia, including several components of the 26S proteasome (e.g. subunit beta type-3, PSMB3; subunit 4, PSMD4; subunit alpha 6, PSMA6; non-ATPase subunit 12, PSMD12; subunit 13, PSMD13) and the COP9 signalosome complex (e.g. subunit 2, COPS2; subunit 3, COPS3 in juvenile hearts; NGG1 interfacing factor 3 like 1, NIF3L1). The 26S proteasome and the COP9 signalosome are highly conserved multi-unit proteolytic complexes that catalyze the controlled degradation of damaged or unnecessary proteins via the ubiquitin-proteasome pathway. Indeed, the ubiquitin-proteasome pathway is known to play a primary role in the degradation of oxidized proteins in mammalian cells^30^, as well as in supporting cold-adaptation in fish^31,32^, ultimately limiting the accumulation of damaged proteins before they can interfere with cellular processes. In contrast, down regulation of protein ubiquination was identified as a candidate disease marker in genetic mouse models of pathological cardiac hypertrophy^33^. Thus, in the absence of a demonstrable shift in other cellular defense mechanisms, alligator hearts may avoid accumulated oxidative damage by increasing the rate by which proteins are recycled, as is suggested by the increased capacity for both proteolysis and protein synthesis. With the advent of modern, high-throughput techniques to quantify protein turnover rates^34^, it is now feasible to test experimentally whether or not the hypoxic cardiac phenotype is indeed globally or selectively regulating protein turnover. For example, stable isotope labeling by amino acids in cell culture (SILAC) workflows, which can be used in conjunction with iTRAQ tags, could inform on the protein expression dynamics of alligator cardiomyocytes grown in primary culture.

### Functional perspectives

Here we describe changes in the alligator cardiac proteome that occur during embryonic development in a hypoxic environment, and carry forward to later life stages even when oxygen is no longer limiting. The dominant question then becomes, do these changes impart a long-term cost or benefit on heart function? We know from previous studies that functional differences in cardiac performance are clearest, and sometimes only visible, when the animal is tested under hypoxic conditions or during periods of high oxygen demand. At the protein level, these functional differences could be driven by phosphorylation and/or post-translational modifications that are not necessarily reflected in changes to steady-state protein abundance, or that only occur under hypoxic conditions. Yet our data clearly demonstrate concerted and lasting changes in protein expression, suggesting that reprogramming the cardiac proteome is an important element of this phenotypic change. As such, the protein signatures of the hypoxic-conditioned heart are expected to confer two major advantages to the alligator by supporting improved cardiac performance during hypoxia (ex. breath-holding during diving) and hypoxemia (ex. during exercise or digestion). First, the enlarged heart will be a more efficient pump for supplying oxygen to tissues. At the proteome level, this is reflected in increased tissue integrity (e.g. structural and adhesion proteins), and the absence of a fibrotic signature that would be indicative of pathological cardiac hypertrophy. A detailed histological analysis of the hypoxic heart phenotype would add weight to these findings. Second, increased mitochondrial efficiency will support energy production in the myocardium during hypoxia/hypoxemia, since there are obvious limitations to metabolic suppression available in the working heart. Mitochondria isolated from juvenile alligator hearts show that developmental hypoxic exposure lowers leak respiration and increases respiratory control ratios^12^. At the protein level, improved mitochondrial efficiency is suggested by the increased expression of electron transport chain components, and any consequent increase in oxidative damage could be mitigated by an increased capacity for protein turnover. It is important to note, however, that protein turnover is itself energetically expensive, and over time this may represent a negative consequence of hypoxia-induced cardiac reprogramming. Alternatively, this added cost could be met by the observed protein-level changes in lipid oxidation – supporting increased preference for this high-yield energy substrate – as well as TCA cycle enzymes to feed mitochondrial oxidative phosphorylation. Quantifying substrate flux through energy pathways in hypoxic-conditioned hearts, under both normoxia and hypoxia, is an important next step.

## Methods

### Animals

American alligator (*Alligator mississippiensis*) eggs were collected from a total of 8 nests at the Rockefeller Wildlife Refuge in Grand Chenier, LA. Eggs were transported to the University of North Texas (Denton, TX) for the study. To establish the initial embryonic age, two eggs from each clutch were used for staging according to Ferguson^35^. All eggs were weighed, numbered, and randomly placed in plastic containers containing a 1:1 vermiculite:water mixture. Embryos were incubated at 30 °C in a walk-in incubation room (Percival Scientific, Perry, IA), ensuring that all embryos developed as females. At approximately 20% of incubation (total incubation time is 72 days at 30 °C) all eggs were randomly assigned to incubation conditions of either 21% oxygen (21% O_2_; normoxia) or 10% oxygen (10% O_2_; hypoxia) as previously described^12,36^. These oxygen percentages were chosen to build on extensive studies previously conducted investigating embryonic alligator development^12,15,37,38^, and the close proximity to a previous measure from a crocodilian nest^9^. Egg containers were sealed inside large Ziploc bags instrumented with an inflow and outflow port for airflow. Air composition was continuously monitored with an oxygen analyzer (S-3AI, Ametek Applied Electrochemistry Pittsburgh, PA, USA).

Two sampling periods were used for the study. At 90% of incubation, four clutch-matched eggs originating from four nests were removed from each condition and euthanized using isoflourane. Body mass was taken and hearts without the major outflow tracts were then extracted and flash frozen. The remaining eggs were returned to 21% O_2_ until hatch, at which point the juveniles were marked by tail scute clipping in order to identify the incubation condition and clutch of origin. All animals were then maintained identically for two years in 0.7 × 2 × 0.7 m fiberglass pens, with free access to water at an ambient temperature that ranged from 24 to 28 °C. The animals were fed commercial alligator food and maintained under a 12 h:12 h light:dark cycle. For cardiac sampling, animals were euthanized via isoflurane ventilation. Body mass was then taken and the whole hearts were extracted, weighed, and flash frozen in liquid nitrogen. The juveniles originated from four nests that did not coincide with the previously sampled embryos; there was one clutch-match between juvenile normoxic and hypoxic conditions, and one nest was represented three times in the hypoxic juvenile condition. All samples were stored at −80 °C until processing. The experiments were approved by the University of North Texas animal ethics committee IACUC (#17-001) in accordance with AWA regulations.

### Protein extraction and iTRAQ labelling

A total of 15 frozen ventricles were used to characterize and quantify the cardiac proteome of alligators at two developmental stages and two levels of oxygen treatment (n = 4 each for embryos in normoxia and hypoxia; n = 4 juvenile normoxia; n = 3 juvenile hypoxia). Frozen hearts were powdered on dry ice with a mortar and pestle, and then 40 mg of tissue was homogenized in 500 μl buffer (100 mM HEPES, 0.1 M DTT, 4% w/v SDS, pH 7.6) containing 1x MS-SAFE Protease and Phosphatase Inhibitors (Sigma-Aldrich, Oakville, ON) using a Precellys 24 and 2 mm zirconium oxide beads (2 × 25 s at 6800 rpm; Bertin Instruments, Montigny-le-Bretonneux, France). To facilitate dissociation of membrane proteins, the crude homogenate was twice heated to 95 °C for 5 min and briefly sonicated on ice, then clarified by centrifugation (13 000 g × 10 min). Proteins in the supernatant fraction were precipitated using the Calbiochem Protein Extraction Kit (EMD Millipore, Billerica, MA) according to manufacturer’s instructions, and reconstituted in 75 μl buffer (1 M HEPES, 8 M urea, 2 M thiourea, 4% w/v CHAPS, pH 8.5). For each sample, 200 μg total protein was transferred to an Amino Ultra-0.5-centrifugation filter device (10 K nominal molecular weight cut-off) that was previously passivated with 5% Tween-20 to increase protein recovery^39^. Samples were washed three times with UA buffer (8 M urea, 0.1 M HEPES, pH 8.5), and then incubated for 20 min in UA buffer containing 0.5 M iodoacetamide (Sigma). After washing three times with 0.5 M triethylammonium bicarbonate (TEAB; Sigma), proteins were digested overnight at 37 °C with 4 μg MS-grade trypsin (Promega Corporation, Madison, WI). Digested peptides were recovered and labelled using two 8-plex iTRAQ kits (SCIEX, Framingham, MA) according to manufacturer’s instructions. One sample (embryo, normoxia) was labelled in replicate reactions to serve as an internal control on the 2 plexes. Labeled peptides were pooled and purified using C18 columns (Sigma), and eluted with 70% acetonitrile containing 0.1% formic acid.

### Separation of peptides and LC mass spectrometric analysis

Analysis of iTRAQ-labelled peptides by mass spectrometry was performed by SPARC BioCenter Molecular Analysis (The Hospital for Sick Children, Toronto, ON) as previously described^40^. Identification and quantification of cardiac proteins was performed using Proteome Discoverer v2.2.0.388 (Thermo-Fisher, Waltham, MA). Mass spectra were searched against the NCBI non-redundant protein database (March 8, 2018) using the following spectrum file search settings: 20 ppm precursor mass tolerance, 0.5 Da fragment mass tolerance, carbamidomethylation and iTRAQ static modifications, at least 2 unique identifying peptides, and target false discovery rate (FDR) 5%. Protein abundances were normalized to total protein in each run and missing values imputed by low abundance re-sampling. Data was scaled to the internal control sample to allow direct comparison between the 2 plexes. Only proteins that were identified on both plexes were considered.

### Bioinformatics analyses

Gene ontology (GO) classification of the alligator cardiac proteome was carried out using Blast2GO. Differentially abundant proteins were identified by two-way ANOVA within the software platform Perseus^41^, with *age* (embryo, juvenile) and *oxygen* (normoxia, hypoxia) as main factors and allowing for their *interaction* (*age* × *oxygen*), with significance level set at p < 0.05. Functional analysis of the differentially abundant proteins, including GO term enrichment, protein-protein interaction (PPI) networks, and Kyoto Encyclopedia of Genes and Genomes (KEGG) pathway analyses, were carried out in STRING (https://string-db.org; September 17, 2018). Visualization of non-redundant GO term functional enrichment was carried out under default settings in REVIGO (http://revigo.irb.hr).

### Cross-validation by quantitative reverse-transcription polymerase chain reaction (qRT-PCR)

The effects of developmental hypoxia exposure on the protein abundance of Natriuretic Polypeptide A (nppa) were opposite in embryonic and juvenile hearts; therefore this protein was selected for cross-validation using qRT-PCR. Briefly, total RNA was extracted from approximately 50 mg of powdered ventricular tissue using Trizol reagent (Invitrogen Life Technologies, Carlsbad, CA), and then 1 μg of total RNA was reverse transcribed to cDNA using the High Capacity Reverse Transcription Kit (Invitrogen), both according to manufacturer’s instructions. Gene expression was quantified in duplicate 12 μl reactions containing 1x Power SYBR Green Master Mix (Invitrogen), 200 nM each forward and reverse primer, and 3 μl diluted cDNA template. Primer3 v.0.4.0 (http://bioinfo.ut.ee/primer3-0.4.0/) was used to design gene-specific primers for *nppa* (fwd: 5′-CATTTCTCTACGGGCTCCTG-3′, rev: 5′-TCCTCAGCTTTAGGCTCCTG-3′; GenBank accession no. NW_017711487). Average threshold cycle values for each sample were fitted to the antilog of standard curves generated from serially diluted cDNA, and normalized to the expression of ribosomal protein L8 (*rpl8*; fwd: 5′-GGTGTGGCTATGAATCCTGT-3′; rev: 5′-ACGACGAGCAGCAATAAGAC-3′^42,43^). Primer specific amplification efficiencies were: *nppa* 109%, *rpl8* 112%.

## Supplementary information

Supplementary Figure S1

Supplementary Data S1

## Data Availability

Data deposited to the ProteomeXchange with the identifier PXD013974.
