# Supplementary material for: Hypoxia-induced reprogramming of the cardiac phenotype in American alligators (Alligator mississippiensis) revealed by quantitative proteomics
Source: Sci Rep. 2019 Jun 13;9:8592. doi: 10.1038/s41598-019-45023-3 (PMC6565670; doi:10.1038/s41598-019-45023-3)
Supplement: Supplementary file 1 — Supplementary Figure S1 [file 41598_2019_45023_MOESM1_ESM.docx]

Supplemental Information for **Hypoxia-induced reprogramming of the cardiac phenotype in American alligators (*Alligator mississippiensis*) revealed by quantitative proteomics**

Sarah L. Alderman^*1^, Dane A. Crossley, II^2^, Ruth M. Elsey^3^ and Todd E. Gillis^1^

^1^Department of Integrative Biology, University of Guelph, Guelph, Ontario, N1G 2W1 Canada

^2^Developmental Integrative Biology Research Group, Department of Biological Sciences, University of North Texas, Denton, Texas, 76203-5017, USA

^3^Louisiana Department of Wildlife and Fisheries, Rockefeller Wildlife Refuge, Grand Chenier, Louisiana, 70643, USA.

*Corresponding author: alderman@uoguelph.ca

**Supplementary Figure S1. Relationship between protein expression (iTRAQ) and gene expression (qRT-PCR) for natriuretic peptide A (nppa).** Data is the average expression for each of the four treatment groups: open symbols are normoxia and closed symbols are hypoxia; diamonds are embryonic hearts and squares are juvenile hearts. Gene expression was normalized to the housekeeping gene *ribosomal protein L8* (*rpl8*), and protein expression was normalized and scaled as described in as described in Materials & Methods.
